# Adolescents’ and Young Adults’ Use and Perceptions of Pod-Based Electronic Cigarettes

**DOI:** 10.1001/jamanetworkopen.2018.3535

**Published:** 2018-10-19

**Authors:** Karma McKelvey, Mike Baiocchi, Bonnie Halpern-Felsher

**Affiliations:** 1 Division of Adolescent Medicine, Department of Pediatrics, Stanford University, Palo Alto, California; 2 Stanford Prevention Research Center, Stanford University, Palo Alto, California

## Abstract

**Importance:**

Electronic cigarettes (e-cigarettes) are the most commonly used tobacco product among adolescents and young adults, and the new pod-based e-cigarette devices may put adolescents and young adults at increased risk for polytobacco use and nicotine dependence.

**Objective:**

To build an evidence base for perceptions of risk from and use of pod-based e-cigarettes among adolescents and young adults.

**Design, Setting, and Participants:**

In a survey study, a cross-sectional analysis was performed of data collected from April 6 to June 20, 2018, from 445 California adolescents and young adults as part of an ongoing prospective cohort study designed to measure the use and perceptions of tobacco products.

**Exposures:**

Use of pod-based e-cigarettes, e-cigarettes, and cigarettes.

**Main Outcomes and Measures:**

Ever use, past 7-day use, and past 30-day use and co-use of pod-based e-cigarettes, e-cigarettes, and cigarettes; use of flavors and nicotine in pod-based e-cigarettes and e-cigarettes; and associated perceptions of risks, benefits, and nicotine dependence.

**Results:**

Among 445 adolescents and young adults (280 females, 140 males, 6 transgender individuals, and 19 missing data; mean [SD] age, 19.3 [1.7] years) who completed wave 6 of the ongoing prospective cohort study, ever use information was provided by 437 respondents, of which 68 (15.6%) reported use of pod-based e-cigarettes, 133 (30.4%) reported use of e-cigarettes, and 106 (24.3%) reported use of cigarettes. The mean (SD) number of days that pod-based e-cigarettes were used in the past 7 days was 1.5 (2.4) and in the past 30 days was 6.7 (10.0). The mean (SD) number of days that other e-cigarettes were used in the past 7 days was 0.8 (1.8) and in the past 30 days was 3.2 (7.4). The mean (SD) number of days that cigarettes were used in the past 7 days was 0.7 (1.8) and in the past 30 days was 3.0 (7.6). Among ever users of pod-based e-cigarettes, 18 (26.5%) reported their first e-liquid was flavored menthol or mint and 19 (27.9%) reported fruit (vs 13 [9.8%] and 50 [37.6%] for other e-cigarettes). The mean perceived chance of experiencing social risks and short-term and long-term health risks from the use of either pod-based e-cigarettes or other e-cigarettes was 40% and did not differ statistically by e-cigarette type. Among 34 adolescents and young adults reporting any loss of autonomy from nicotine, there was no difference in mean (SD) Hooked On Nicotine Checklist scores between those using pod-based e-cigarettes (2.59 [3.14]) and other e-cigarettes (2.32 [2.55]).

**Conclusions and Relevance:**

Use by adolescents and young adults of newer types of e-cigarettes such as pod-based systems is increasing rapidly, and adolescents and young adults report corresponding misperceptions and lack of knowledge about these products. Rapid innovation by e-cigarette manufacturers suggests that public health and prevention efforts appear to be needed to include messages targeting components common to all current and emerging e-cigarette products to increase knowledge and decrease misperceptions, with the goal to try to ultimately reduce e-cigarette use among adolescents and young adults.

## Key Points

**Question:**

How and why are California adolescents and young adults using new pod-based electronic cigarettes (e-cigarettes)?

**Findings:**

This survey study of 445 adolescents and young adults revealed similar chances (40%) of experiencing negative health and social consequences from using pod-based and/or other types of e-cigarettes. Among 34 adolescents and young adults reporting any loss of autonomy from nicotine, there was no difference in mean Hooked On Nicotine Checklist scores between those using pod-based and other e-cigarettes.

**Meaning:**

Increasing use of pod-based e-cigarettes among otherwise nicotine-naive adolescents and young adults could be associated with the absence of clear, consistent public health warnings and messaging targeting relevant aspects common to all types of e-cigarettes.

## Teaser

This survey study examines the perceptions of risk from and use of pod-based electronic cigarettes (e-cigarettes) among adolescents and young adults.

## Introduction

Electronic cigarettes (e-cigarettes) are the most commonly used tobacco product among adolescents and young adults.^1^ Liquids aerosolized by e-cigarettes (e-liquids) typically contain flavorings, moisture-retaining substances (ie, propylene glycol or glycerol), and nicotine.^2^ Around the world, there is seemingly ceaseless demand by adolescents and young adults for novel and improved “high-tech” products, understood among this group to impart status.^3,4^ E-cigarettes are such devices for the adolescents and young adults who use them,^5,6^ with device modification adding to their appeal.^7,8^ The availability of e-liquids in a multitude of flavors^9^ and favorable perceptions of harm associated with e-cigarettes,^3,4^ including e-cigarettes being considered the least harmful of all tobacco products,^9,10,11^ have also contributed to the rapid global adoption of e-cigarettes by adolescents and young adults. Although evidence of the health risks of e-cigarettes is nascent,^12^ ample evidence exists that exposure to nicotine by adolescents and young adults is associated with myriad health risks,^1,12,13,14,15,16^ and the use of e-cigarettes increases the odds of initiation of smoking traditional tobacco cigarettes.^17,18,19^ Exemplifying concern with youth uptake and use of e-cigarettes in general and pod-based e-cigarettes in particular, the US Food and Drug Administration released a statement in April 2018 on new enforcement actions and a Youth Tobacco Prevention Plan to stop youth use of, and access to, JUUL (brand name for a pod-based e-cigarette) and other e-cigarettes.^20^

In contrast to the comparatively stagnant market for traditional cigarettes, the e-cigarette market allows for rapid product innovation by manufacturers. Accordingly, in June 2015 a new pod-based style of e-cigarette (the JUUL) became available. Although pod-based e-cigarettes aerosolize flavored e-liquids like other brands of e-cigarettes,^21,22^ there are important differences between the types of e-cigarettes. JUUL e-cigarettes, conceived in an innovative design school program and marketed heavily as an alternative to cigarettes, were, until recently, available in only 1 nicotine concentration: 59 mg/mL. According to the company’s website: “Each JUULpod is designed to contain approximately 0.7 mL with 5% nicotine by weight at time of manufacture which is approximately equivalent to 1 pack of cigarettes or 200 puffs.”^23^ Also, unlike other types of e-cigarettes that require users to add e-liquid, purchased separately, to the reservoir of the device, pod-based e-cigarettes use USB-shaped prefilled pods. Furthermore, the sleek, small, and “high-tech” look of pod-based e-cigarettes makes it easy to hide the e-cigarettes in one’s hand and may not appear at first glance, or to those unfamiliar with the design, to even be an e-cigarette.^24^ Sales of JUULs have increased quickly, and their market share was listed at 68% of the US e-cigarette market as of July 2, 2018, a 783% increase from the year prior ending June 16, 2018.^25^

Given the novelty, popularity, and potential for harm of pod-based e-cigarettes, data describing how adolescents and young adults use and perceive pod-based devices are both timely and important. Thus far, 1 scientific study has reported data on the use and perceptions of JUULs among US adolescents and young adults aged 15 to 24 years.^26^ That study’s data, collected November 2017, showed that 25% of the sample recognized the JUUL brand, of whom only 25% knew all JUULs contained nicotine. Furthermore, 10% reported ever use of JUULs and 8% reported past 30-day use. Levels of recognition and use of JUULs were higher among those aged 18 to 24 years compared with those aged 15 to 17 years.^26^ Further illustrating the popularity of pod-based e-cigarettes among adolescents and young adults, a study published in April 2018 reported on JUUL-related messages online, including on Reddit (a popular discussion forum).^24^ Authors found, among other things, a “subreddit” (focused discussion group within Reddit) called “UnderageJuul,” which existed from July 2017 until it was banned in January 2018, at which point there were nearly 1000 members. Reported exchanges between members of the UnderageJuul subreddit included identification of retailers not requiring age verification, users of legal age offering to order the product for those who were underage (for a small fee), and sharing strategies about how not to get caught using pod-based e-cigarettes in places where e-cigarette use is banned.^24^ Despite limited empirical data,^27^ numerous articles in the popular press have highlighted the marketing^28^ and popularity of pod-based e-cigarettes.^28,29,30,31^

More scientific data are needed to explicate the effect of this new form of e-cigarettes on the attitudes, initiation, and use of tobacco products among adolescents and young adults. We examine the use of pod-based e-cigarettes and compare it with the use of other e-cigarettes and conventional cigarettes among California adolescents and young adults. We also examine how these pod-based e-cigarettes and other e-cigarettes are used, including flavor choices, perceptions of associated risks and benefits, and symptoms of nicotine dependence. These findings contribute to the basis for future studies to determine how innovation in e-cigarettes affects patterns of tobacco use and may diminish the efficacy of public health tobacco prevention campaigns, which could translate to higher risk for adolescents and young adults using tobacco products. The findings will also inform regulation, marketing, and prevention messaging for e-cigarettes, including the newer pod-based systems.

## Methods

### Participants

The data in this study are derived from wave 6 of an ongoing prospective cohort study of adolescents and young adults recruited from 10 racially/ethnically and socioeconomically diverse high schools across California. From July 13, 2014, through October 11, 2015, all 9th- and 12th-grade students in these schools were invited to enroll and complete the wave 1 survey; of 1299 students who returned consent and assent forms, 772 (59.4%) completed wave 1. The wave 6 sample (n = 445; mean [SD] age, 19.3 [1.7] years; 280 females, 140 males, 6 transgender individuals, and 19 missing data) had more females and a higher percentage of Asian students than the schools from which we recruited. Still, participant demographics reflected the demographics of their respective schools. The 5 largest racial/ethnic backgrounds were white (163 [36.6%]), Asian or Pacific Islander (122 [27.4%]), and Hispanic (166 [37.3%]), with 128 (28.8%) nonwhite Hispanic individuals and 38 (8.5%) white Hispanic individuals (Table 1). The survey and research protocol were approved by the Stanford University Institutional Review Board. Consent forms, assent forms, and project information sheets were given to students to take home and review with their parents or guardians. Prospective participants provided signed parental informed consent and assent forms; students 18 years or older provided their own written informed consent. This study followed the American Association for Public Opinion Research (AAPOR) reporting guideline.

**Table 1.** Comparison of Participant Demographics Between Wave 1 (2014-2015) and Wave 6 (2018)^a^

Covariate	Participants, No. (%)		χ^2^ for Difference	*P* Value
	Wave 1 (n = 772)	Wave 6 (n = 445)		
Sex				
Female	483 (62.6)	280 (62.9)	12	.21
Male	277 (35.9)	140 (31.5)		
Transgender	1 (0.1)	6 (1.3)		
Missing	11 (1.4)	19 (3.4)		
Grade at study start				
Freshman	311 (40.3)	181 (40.7)	15	.24
Sophomore	2 (0.3)	2 (0.4)		
Junior	10 (1.3)	6 (1.3)		
Senior	439 (56.9)	251 (56.4)		
Missing	10 (1.3)	5 (1.1)		
Race				
American Indian or Alaskan Native	18 (2.3)	8 (1.8)	48	.24
Asian	150 (19.4)	113 (25.4)		
Native Hawaiian or Pacific Islander	23 (3.0)	9 (2.0)		
Black or African American	27 (3.5)	9 (2.0)		
White	271 (35.1)	163 (36.6)		
More than 1 race	191 (24.7)	99 (22.2)		
Not sure	32 (4.1)	19 (4.3)		
Race missing	60 (7.8)	25 (5.6)		
Ethnicity				
Hispanic or Latino	316 (40.9)	166 (37.3)	12	.21
Not Hispanic or Latino	411 (53.2)	261 (58.7)		
Not sure	21 (2.7)	13 (2.9)		
Ethnicity missing	24 (3.1)	5 (1.1)		
Born in the United States				
Yes	669 (86.7)	386 (86.7)	6	.20
No	90 (11.7)	53 (11.9)		
Missing	13 (1.7)	6 (1.3)		
English primary language at home				
Yes	536 (69.4)	315 (70.8)	6	.20
No	224 (29.0)	125 (28.1)		
Missing	12 (1.6)	5 (1.1)		

^a^ Analyses for this study were constrained to wave 6 data.

### Design and Setting

Participants received a unique log-in identification to complete an online survey, administered by Qualtrics. In recognition of the entry of JUUL e-cigarettes (and, recently, more brands of pod-based e-cigarettes) into the market and their rapidly increasing market share, we added questions using the term “JUUL,” defined in our survey as follows: “A JUUL is an electronic vaping device that uses nicotine salts (crystals) to deliver a nicotine aerosol…” to our wave 6 survey, with many questions mirrored from other published measures and findings concerning e-cigarettes and other tobacco products.^9,10^ Data for wave 6 were collected from April 6 to June 20, 2018. Additional details regarding the study design, data collection, and sampling are published elsewhere.^9,32^ Participants received a $35 gift card for completing wave 6.

### Main Outcomes

Measures are briefly summarized below. Survey questions and response choices reported on in this study are provided verbatim in the eAppendix in the Supplement.

#### Tobacco Products

Prior to answering questions about tobacco products, participants viewed pictures and descriptions of cigarettes, e-cigarettes, and JUULs to ensure that participants focused on the same products. This is standard practice for surveys about e-cigarettes.^33,34^ To separate JUULs from other e-cigarettes, questions about e-cigarettes were identified using the language: “e-cigarettes/vapes (not including JUULs).”

#### Recognition

The survey asked participants, “Before today have you ever heard of a JUUL?” The response choices were yes or no. Participants were then asked about use, nicotine dependence, flavors of e-liquid, social norms, acceptability, perceived prevalence of use, and perceptions of risks and benefits separately for pod-based e-cigarettes, other e-cigarettes, and cigarettes.

#### Use

Participants were asked about ever use and number of days used in the past 7 days and past 30 days. Ever users of any type of e-cigarette were asked about the first type of e-cigarette they used.

#### Nicotine Dependence

The Hooked on Nicotine Checklist (HONC), a validated measure of the severity of reduced autonomy from nicotine, was used (HONC scores range from 0 to 10).^35^ Endorsement of any symptom indicates loss of full autonomy, and increasing scores indicate more severe loss of autonomy, presumed to signify greater nicotine dependence.

#### Flavors

Participants were asked if their first e-liquid was flavored; if the response was yes, they were asked what the first flavor was that they used. Response choices for JUUL e-cigarettes reflected available JUULpod flavors at the time of survey administration: not sure/don’t remember, mango, cool mint, Virginia tobacco, fruit medley, crème brûlée, cool cucumber, classic tobacco, cool menthol, and other (participants could fill in the flavor). For comparability and ease of interpretation, flavors were categorized into the following: tobacco, menthol and mint, fruit, dessert and sweets, alcohol, spice, candy, coffee and tea, beverage, unflavored, and don’t know and other, as delineated by Yingst et al.^36^

#### Social Norms, Acceptability, and Perceived Prevalence

Participants were asked how many of their 5 closest friends and how many of 100 adolescents and young adults their age have tried e-cigarettes or cigarettes, used e-cigarettes or cigarettes in the past 30 days, and use e-cigarettes or cigarettes regularly? They were then asked to indicate their agreement with statements that their friends think it is okay to try e-cigarettes or cigarettes, use e-cigarettes or cigarettes once in a while, or use e-cigarettes or cigarettes regularly.

#### Perceptions of Risks and Benefits

Queries regarding risks and benefits of e-cigarettes or cigarettes presented a scenario describing a specific context to consider. After the scenario, participants were asked the perceived chance (0%-100%) of experiencing specific health and social risks and benefits (eTable 1 in the Supplement).

### Study Size and Potential Bias

There has been dropout across the waves of the study, and the original sampling frame was all students in the 9th and 12th grades from the 10 participating high schools. Rather than make population-level estimates beyond these schools, the overarching cohort study was designed to examine changes in use and perceptions of tobacco products over time. The current analysis is constrained to wave 6, the only survey with items pertaining to pod-based e-cigarettes. In accordance with the AAPOR reporting guideline for survey studies,^37^ the participation rate for wave 6 was 43.0% (540 students who initiated the survey of 1257 viable e-mail invitations containing survey links). Data for this study included only participants who completed the wave 6 survey (445 [82.4%]). Although there were differences in proportions for race/ethnicity between waves 6 and 1, no adjustments for dropouts are reported, as previous work with this cohort has revealed no association between race/ethnicity and our outcomes of interest.^9,10,32^

### Statistical Analysis

Descriptive summaries include counts, means, and percentages. Standard deviations were created using bootstrap estimates, which accounted for school clustering; no weighting scheme was deployed.

Because of the well-established difference in adolescent perceptions between cigarettes and e-cigarettes, cigarettes were included only in summaries of use.^10,38^ Wilcoxon signed rank tests were performed to assess within-subject use patterns in the past 7 days and the past 30 days for pod-based and other e-cigarettes.

Exploratory analyses of HONC scores were performed. Ever users of any e-cigarettes were classified as either reporting signs of diminished autonomy (HONC score 1) or full autonomy (HONC score 0). A McNemar test was then performed comparing full autonomy with loss of autonomy. Next, to summarize the association between autonomy from nicotine and participants’ age and sex, for both products, the binary outcome (diminished autonomy or full autonomy) was regressed on age (continuous) and sex (categorical). The Wilcoxon rank sum test was used to compare mean HONC scores by product. Among only those who reported diminished autonomy, mean HONC scores were compared by product using a paired *t* test. All *P* values were from 2-sided tests and results were deemed statistically significant at *P* < .05.

## Results

### Recognition and Use

A total of 229 of 445 students (51.5%) in our sample had heard of JUUL e-cigarettes, which did not significantly vary by sex. Ever use information was provided by 437 respondents, of which 68 (15.6%) reported use of pod-based e-cigarettes, 133 (30.4%) reported use of other e-cigarettes, and 106 (24.3%) reported use of cigarettes. The proportions of students reporting past 7-day use and past 30-day use of pod-based e-cigarettes, e-cigarettes, and cigarettes, together with frequencies of use among ever users, are shown in Table 2. Wilcoxon signed rank tests for differences detected differences between pod-based e-cigarettes and cigarettes for both past 7-day use (Wilcoxon statistic, 127; *P* = .02) and past 30-day day use (Wilcoxon statistic, 161.5; *P* = .04); differences between pod-based e-cigarettes (Wilcoxon statistic, 238; *P* = .21) and other e-cigarettes (Wilcoxon statistic, 143; *P* = .41) were not significant. The mean (SD) number of days that other e-cigarettes were used in the past 7 days was 0.8 (1.8) and in the past 30 days was 3.2 (7.4). The mean (SD) number of days that cigarettes were used in the past 7 days was 0.7 (1.8) and in the past 30 days was 3.0 (7.6). Among ever users of pod-based e-cigarettes, 18 (26.5%) reported their first e-liquid was flavored menthol or mint and 19 (27.9%) reported fruit (vs 13 [9.8%] and 50 [37.6%] for other e-cigarettes).

**Table 2.** Use of Pod-Based e-Cigarettes, Other e-Cigarettes, and Cigarettes Among 437 California Adolescents and Young Adults in 2018^a^

Product	Ever Use, No. (%)	Any Past 30-d Use, No./Total No. (%)	Past 30-d Use, Mean (SD), d	Any Past 7-d Use, No./Total No. (%)	Past 7-d Use, Mean (SD), d
Pod-based e-cigarettes	68 (15.6)	40/68 (58.8)	6.7 (10.0)	25/68 (36.8)	1.5 (2.4)
Other e-cigarettes	133 (30.4)	40/133 (30.1)	3.2 (7.4)	26/133 (19.5)	0.8 (1.8)
Cigarettes	106 (24.3)	30/106 (28.3)	3.0 (7.6)	17/106 (16.0)	0.7 (1.8)

^a^ Mean (SD) age, 19.3 (1.7) years.

For users of pod-based e-cigarettes, co-use of other e-cigarettes and cigarettes was high, with only 4 of 163 students (2.5%) reporting exclusive use of pod-based e-cigarettes (vs 33 of 163 [20.2%] for exclusive use of other e-cigarettes and 24 of 163 [14.7%] for cigarettes) (Table 3); 41 of 163 participants (25.2%) reported use of all 3 products. Most often, pod-based e-cigarette users’ first-used form of e-cigarette was a “vape pen” (14 of 61 [23.0%]), followed by “mods” (8 of 61 [13.1%]) and “large size ‘tank’ device” (8 of 61 [13.1%]), with 6 of 61 participants (9.8%) reporting pod-based e-cigarettes as their first form of e-cigarette. Of the 132 users of other e-cigarettes, 39 (29.5%) indicated that a “vape pen” was their first form of e-cigarette, followed by “disposable/single use e-cigarettes” (18 [13.6%]), with 6 participants (4.5%) reporting JUUL as their first form of e-cigarette (these are the same 6 respondents cited in the use summary for pod-based e-cigarettes).

**Table 3.** Frequency and Proportion of Participants Reporting Ever Use of Pod-Based e-Cigarettes, e-Cigarettes, and/or Cigarettes Among 445 California Adolescents and Young Adults in 2018^a^

Tobacco Product	Participants, No. (%) (n = 163)
Pod-based e-cigarettes only	4 (2.5)
e-Cigarettes only	33 (20.2)
Cigarettes only	24 (14.7)
Pod-based e-cigarettes and e-cigarettes only	21 (12.9)
Pod-based e-cigarettes and cigarettes only	2 (1.2)
e-Cigarettes and cigarettes only	38 (23.3)
Pod-based e-cigarettes, e-cigarettes, and cigarettes	41 (25.2)

Abbreviation: e-cigarette, electronic cigarette.

^a^ Mean (SD) age, 19.3 (1.7) years.

### Nicotine Dependence

Models used to summarize associations between the loss of autonomy from nicotine and the variables of sex and age showed very little association with sex (all estimates were close to null) (eTable 2 in the Supplement shows regression results). A McNemar test considering any loss of autonomy vs some loss of autonomy revealed no significant within-participant differences between ever users of pod-based e-cigarettes vs ever users of other e-cigarettes (McNemar χ^2^_1_ = 1.07; *P* = .30). A Wilcoxon signed rank test of the distributions of HONC scores among ever users similarly showed no statistically significant difference (*V* = 123; *P* = .29). Among participants who reported any loss of autonomy (n = 34), there was no difference in mean (SD) HONC scores between pod-based e-cigarettes (2.59 [3.14]) and other e-cigarettes (2.32 [2.55]; *t*_33_ = 0.48; *P* = .63).

### Use of Flavors

Among ever users of pod-based e-cigarettes, 48 of 65 (73.8%) indicated that their first pod was flavored, and 17 of 65 (26.2%) indicated that theirs was not flavored. Of 65 ever users of pod-based e-cigarettes, the largest proportion (22 [34.9%]) reported that they were not sure or did not remember their first flavor, 18 (28.6%) reported cool mint or menthol as the first flavor, 19 (30.2%) reported fruit, 3 (4.8%) reported desserts or sweets, and 1 (1.6%) reported classic tobacco (Table 4). Among users of pod-based e-cigarettes, menthol or mint (18 [28.6%]) and fruit (19 [30.2%]) flavor categories were the most-reported first flavors; 50 of 107 (46.7%) users of other e-cigarettes reported fruit as their first flavor. No participant indicated using a flavor from the categories of alcohol, nuts or spices, candy, beverage, or unflavored (Table 4).

**Table 4.** First Flavors Used by Participants Reporting Use of Pod-Based e-Cigarettes and Other e-Cigarettes Among 445 California Adolescents and Young Adults in 2018^a^

First Flavor Used	Participants, No./Total No. (%)	
	Pod-Based e-Cigarettes	Other e-Cigarettes
First product flavored (yes)	48/65 (73.8)	108/133 (81.2)
First flavor used		
Tobacco	1/63 (1.6)	1/107 (0.9)
Menthol or mint	18/63 (28.6)	13/107 (12.1)
Fruit	19/63 (30.2)	50/107 (46.7)
Dessert or sweets^b^	3/63 (4.8)	19/107 (17.8)
Coffee or tea	0	1/107 (0.9)
Don’t know or other	22/63 (34.9)	23/107 (21.5)

Abbreviation: e-cigarette, electronic cigarette.

^a^ Mean (SD) age, 19.3 (1.7) years.

^b^ Our survey response category “candy or dessert flavors (eg, caramel, vanilla, chocolate, ice cream, mud pie)” precluded us from differentiating between “candy” and “dessert.”

### Social Norms (Use by Friends and Perceived Prevalence and Acceptability)

Participants reported that, of their 5 closest friends, on average, just over 2 had tried pod-based e-cigarettes, 3 had used pod-based e-cigarettes in the past 30 days, and 2 regularly use pod-based e-cigarettes. These numbers are virtually identical to those for use of other e-cigarettes (Table 5). Participants believed that, among 100 adolescents and young adults their age, 38.4 had tried pod-based e-cigarettes, and 30.6 had used pod-based e-cigarettes in the past 30 days or used regularly; numbers were slightly higher for other e-cigarettes (Table 5). Of 336 respondents, a total of 70 participants (20.8%) strongly agreed that it is okay to try pod-based e-cigarettes and 94 (28.0%) strongly disagreed, 76 (22.6%) strongly agreed it is okay to use pod-based e-cigarettes once in a while and 104 (31.0%) strongly disagreed, and 19 (5.7%) strongly agreed it is okay to use pod-based e-cigarettes regularly and 128 (38.1%) strongly disagreed. Proportions were similar for other e-cigarettes (eTable 3 in the Supplement).

**Table 5.** Comparison of Use of Pod-Based e-Cigarettes and Other e-Cigarettes Among 445 California Adolescents and Young Adults in 2018^a^

Product	Tried	Friends Who Used in Past 30 d	Use Regularly
Among 5 closest friends, mean (SD), No.			
Pod-based e-cigarettes	2.4 (1.1)	3.0 (1.5)	2.0 (0.9)
Other e-cigarettes	2.5 (1.1)	3.1 (1.5)	2.1 (1.0)
Among 100 people their age, mean (SD), No.			
Pod-based e-cigarettes	38.4 (28.0)	30.6 (26.0)	29.8 (25.8)
Other e-cigarettes	45.5 (24.7)	38.2 (24.7)	36.0 (24.6)

Abbreviation: e-cigarette, electronic cigarette.

^a^ Mean (SD) age, 19.3 (1.7) years.

### Perceptions of Risks and Benefits 

On average, participants perceived a 40% chance of experiencing social risks and short-term and long-term health risks from using pod-based e-cigarettes. Other than the lower perceived mean (SD) chance of experiencing “other tobacco-related disease” using pod-based e-cigarettes (44.0 [34.2]) vs e-cigarettes (48.7 [33.5]; *t*_888_ = 2.07; *P* = .04), the perceived chance of experiencing each risk and benefit was comparable for both types of e-cigarettes, although greater variability was observed for non-pod-based e-cigarettes for the item “better concentration” (eTable 1 in the Supplement).

## Discussion

The 3 most concerning insights from the data presented here are the high prevalence of co-use and polyuse of pod-based e-cigarettes with other e-cigarettes and traditional cigarettes and the higher proportion of participants who reported past 30-day use together with the much higher frequency of use reported for pod-based e-cigarettes vs other e-cigarettes. These findings point to the potential for greatly increased harm for adolescents and young adults associated with the use of pod-based e-cigarettes, as their still-forming brains are particularly vulnerable to the effects of nicotine, with increased earlier exposure to nicotine being associated in a dose-response manner with deleterious health effects.^39^

Our findings of high proportions of students reporting past 30-day use and higher frequency of use for pod-based e-cigarettes align with those of Willett et al.^26^ Emergent evidence of larger proportions of adolescents and young adults reporting past 30-day use of pod-based e-cigarettes suggests a different pattern of uptake among this demographic when one considers the national use pattern of other and earlier styles of e-cigarettes, wherein smaller proportions of users reported past 30-day use.^40^ The high use frequency, prevalent co-use and polyuse of other e-cigarettes and/or traditional cigarettes, and larger proportion of current users of pod-based e-cigarettes could suggest a risk profile dissimilar to that for other e-cigarettes. Furthermore, Willett and colleagues^26^ found that just 37% of current users of pod-based e-cigarettes knew that the product always contains nicotine. This finding shows how changing the form of delivery (eg, pod-based devices vs traditional cigarettes or other e-cigarettes) can confuse user perceptions (eg, nicotine content). Given its addictive nature, nicotine is a stable and worthwhile target for public health prevention messaging.

Although this descriptive study was not prospectively powered for testing group differences, point estimates suggest that adolescents and young adults perceive pod-based e-cigarettes to be less harmful than other e-cigarettes overall, a finding important to clinicians and public health professionals who have occasion to talk with and educate adolescents and young adults. For example, among those reporting any loss of autonomy from nicotine, slightly more severe loss of autonomy was reported by users of pod-based e-cigarettes. In other words, pod-based e-cigarettes are perceived as posing less harm or addictive potential while more nicotine dependence is being reported. This finding exemplifies the disconnect between the harm adolescents and young adults perceive and the harm they report experiencing (here, nicotine dependence), illustrating their apparent inability to connect the idea of addiction to the experience of it.^41^

Moreover, among adolescents and young adults who use them, pod-based e-cigarettes are synonymous with the brand-name JUUL and use is termed “juuling,” whereas “vaping” has typically been used by youths to refer to using all other types of e-cigarettes. This use of language raises concern that adolescents and young adults who use pod-based e-cigarettes could be missed in surveys asking only about “vaping.”^26,27,31^ Although, as with cigarettes and non-pod-based e-cigarettes, mint and menthol and fruit flavors (known to be popular with youths) were the most often-reported flavors first tried,^8,21,42,43,44,45,46^ we also found that many adolescents and young adults were either unaware of the fact that their e-liquid pod was flavored (all pod-based e-cigarette pods are flavored) or were unaware of the flavor that they used. Confusion about flavors could reflect the fact that adolescents typically share their e-cigarettes, regardless of style.^47^

### Limitations

This study has some limitations. The survey draws from schools in California; as diffusion of newer pod-based e-cigarettes likely differs across states, generalization of use patterns is not warranted. Survey questions about pod-based e-cigarettes were not designed to address all the differences in participant understanding of the devices or the JUUL brand; however, questions concerning other e-cigarettes were identified with the phrase “e-cigarettes/vapes (not including JUUL).” We suspect that some differences in perception were obscured by the close proximity in which questions about pod-based e-cigarettes and other e-cigarettes were presented in the survey, although questions were randomly ordered, reducing order bias. Questions differentiated between JUUL and all other e-cigarettes; since other brands of pod-based e-cigarettes are available, it is possible that some respondents reported use of pod-based e-cigarettes that were not the JUUL brand in the category of “e-cigarette/vapes (not JUUL).” Given the primacy of the JUUL brand, it is unlikely that this issue would alter interpretation of the results.

## Conclusions

Although pod-based e-cigarettes are the focus of this study, a broader concern is the emerging dynamic between novel tobacco products such as e-cigarettes and public health prevention efforts. For example, consider how, for years, traditional cigarettes had a clear, unified form for their delivery system and public health messaging was able to target and affect all brands of cigarettes simultaneously. In contrast, e-cigarettes take many forms, with variations seen in devices, flavors, e-liquids, power or voltage, and nicotine content. Hence, amid rapid innovations in e-cigarettes (eg, newer pod-based styles) and in the absence of clear, consistent public health prevention messaging that targets relevant aspects common to all forms of e-cigarettes, further misperception of health risks, including nicotine addiction, could result. Public health prevention efforts are challenged to develop multifaceted and adaptive messaging that is necessary to reduce uptake and consumption of pod-based e-cigarettes and other emergent products among adolescents and young adults who otherwise would likely remain nicotine naive.

## Notes

Supplement. **eTable 1.** Perceived Chance of Experiencing Social and Short- and Long-term Health Risks and Benefits Among 445 California Adolescents and Young Adults in 2018: Comparison Between Use of JUUL and Other Styles of e-Cigarettes **eTable 2.** Results From Logistic Regression Models Summarizing the Relationship Between Participants’ Age and Gender With Autonomy Over Nicotine (Loss of Autonomy/No Loss of Autonomy): Three Models Comparing Between Use of JUUL/Pod-based and Other Styles of e-Cigarettes, Among California Adolescents and Young Adults in 2018 Who Indicated Some Use of the Given Product Type **eTable 3.** Comparison of Perceived Acceptability of Different Use Frequencies Between JUUL and Other Styles of e-Cigarettes Among 445 California Adolescents and Young Adults in 2018 **eAppendix.** Survey Questions
