# Supplementary material for: Adolescents’ and Young Adults’ Use and Perceptions of Pod-Based Electronic Cigarettes
Source: JAMA Netw Open. 2018 Oct 19;1(6):e183535. doi: 10.1001/jamanetworkopen.2018.3535 (PMC6324423; doi:10.1001/jamanetworkopen.2018.3535)
Supplement: Supplement. — eTable 1. Perceived Chance of Experiencing Social and Short- and Long-term Health Risks and Benefits Among 445 California Adolescents and Young Adults in 2018: Comparison Between Use of JUUL and Other Styles of e-Cigarettes eTable 2. Results From Logistic Regression Models Summarizing the Relationship Between Participants’ Age and Gender With Autonomy Over Nicotine (Loss of Autonomy/No Loss of Autonomy): Three Models Comparing Between Use of JUUL/Pod-based and Other Styles of e-Cigarettes, Among California Adolescents and Young Adults in 2018 Who Indicated Some Use of the Given Product Type eTable 3. Comparison of Perceived Acceptability of Different Use Frequencies Between JUUL and Other Styles of e-Cigarettes Among 445 California Adolescents and Young Adults in 2018 eAppendix. Survey Questions [file jamanetwopen-1-e183535-s001.pdf]

Supplementary Online Content

McKelvey K, Baiocchi M, Halpern-Felsher B. Adolescents' and young adults' use and perceptions of pod-based electronic cigarettes. *JAMA Netw Open.* 2018;1(6):e183535. doi:10.1001/jamanetworkopen.2018.3535

eTable 1. Perceived Chance of Experiencing Social and Short- and Long-term Health Risks and Benefits Among 445 California Adolescents and Young Adults in 2018: Comparison Between Use of JUUL and Other Styles of e-Cigarettes
eTable 2. Results From Logistic Regression Models Summarizing the Relationship Between Participants' Age and Gender With Autonomy Over Nicotine (Loss of Autonomy/No Loss of Autonomy): Three Models Comparing Between Use of JUUL/Pod-based and Other Styles of e-Cigarettes, Among California Adolescents and Young Adults in 2018 Who Indicated Some Use of the Given Product Type
eTable 3. Comparison of Perceived Acceptability of Different Use Frequencies Between JUUL and Other Styles of e-Cigarettes Among 445 California Adolescents and Young Adults in 2018
eAppendix. Survey Questions

This supplementary material has been provided by the authors to give readers additional information about their work.

© 2018 McKelvey K et al. *JAMA Network Open.*

eTable 1. Perceived Chance of Experiencing Social and Short- and Long-term Health Risks and Benefits Among 445 California Adolescents and Young Adults* in 2018: Comparison Between Use of JUUL and Other Styles of e-Cigarettes

JUST BEGAN USING					
	**JUUL**		**Other types**		**x^2 for dif.**
	Mean	SD	Mean	SD	*p*-value
**Social risks**					
Friends will be upset	49.8	41.1	51.5	40.8	0.55
Get into trouble	52.7	40.9	52.8	41.0	0.98
Look cool	16.2	27.4	14.9	26.4	0.51
**Short term health risks**					
Bad cough	50.9	32.9	53.0	32.5	0.34
Trouble catching breath	50.6	33.6	54.3	32.2	0.11
Feel high or buzzed	49.9	36.9	48.8	43.2	0.69
**Long term health risks**					
COPD	21.3	27.5	22.1	27.5	0.66
Become addicted	60.8	33.9	62.7	32.0	0.41
**Benefits**					
Less stressed	26.9	29.5	27.4	29.6	0.80
Better concentration	10.0	20.6	11.8	45.7	0.47
Less anxious	26.2	29.3	26.9	29.2	0.76
Less depressed	19.9	26.4	21.0	27.0	0.57
**CONTINUE TO USE FOR THE REST OF YOUR LIFE**					
	**JUUL**		**Other types**		**x^2 for dif.**
	Mean	SD	Mean	SD	*p*-value
**Health risks**					
Oral cancer	45.4	32.0	47.1	31.6	0.43
Heart attack	41.8	32.5	42.6	32.0	0.72
Lung cancer	47.1	33.4	49.5	32.2	0.31
Other tobacco related disease	44.0	34.2	48.7	33.5	0.05
Other tobacco related death	42.2	34.2	45.6	33.7	0.16
Wrinkles	51.4	36.7	53.7	35.9	0.37
**Benefits**					
Less anxious	21.0	28.3	20.3	28.0	0.74
Less depressed	48.3	31.9	49.0	30.8	0.74

*mean age 19.3, SD=1.68

© 2018 McKelvey K et al. *JAMA Network Open.*

eTable 2. Results From Logistic Regression Models Summarizing the Relationship Between Participants' Age and Gender With Autonomy Over Nicotine (Loss of Autonomy/No Loss of Autonomy): Three Models Comparing Between Use of JUUL/Pod-based and Other Styles of e-Cigarettes, Among California Adolescents and Young Adults* in 2018** Who Indicated Some Use of the Given Product Type

	**JUUL (N=67)**		**E-CIG (N = 130)**		**EITHER (N=136)**	
	**Coef †**	**p-value**	**Coef †**	**p-value**	**Coef †**	**p-value**
**Age**	0.01	0.98	-0.10	0.64	-0.03	0.86
**Male**	0.22	0.87	-0.23	0.79	-0.45	0.59
**Trans-gender**	-1.45	1.00	-14.1	0.99	-14.24	0.99

* mean age 19.3, SD=1.68
**For both pod-based and other e-cigarettes the binary outcome (diminished autonomy/full autonomy) was regressed on age (continuous) and gender (categorical).
†Coefficients are reported on logit scale.

© 2018 McKelvey K et al. *JAMA Network Open.*

eTable 3. Comparison of Perceived Acceptability of Different Use Frequencies Between JUUL and Other Styles of e-Cigarettes Among 445 California Adolescents and Young Adults* in 2018

FRIENDS THINK IT IS OK TO:	STRONGLY AGREE		AGREE		DISAGREE		STRONGLY DISAGREE	
	JUUL	Other types	JUUL	Other types	JUUL	Other types	JUUL	Other types
TRY	84<br>19.4%	86<br>20.0%	137<br>31.7%	162<br>37.6%	92<br>21.3%	71<br>16.5%	119<br>27.5%	112<br>26.0%
USE ONCE IN A WHILE	100<br>23.4%	99<br>23.1%	64<br>15.0%	62<br>14.5%	134<br>31.3%	150<br>35.0%	130<br>30.4%	118<br>27.5%
USE REGULARLY	26<br>6.0%	32<br>7.4%	99<br>23.0%	114<br>26.4%	147<br>34.2%	131<br>30.3%	158<br>36.7%	155<br>35.9%

*mean age 19.3, SD=1.68

© 2018 McKelvey K et al. *JAMA Network Open.*

eAppendix. Survey Questions
Complete list of survey questions, including response choices, by content area, for measures reported on in this study.

Recognition

Participants were asked: “Before today have you ever heard of a JUUL?” [Response choices: yes or no]

Use

Ever use

Participants were asked: During your entire life, about how many times have you EVER [used a JUUL, used an e-cigarette, smoked a cigarette] even just a puff... [Response choices: Never; 1-2 times; 3-10 times; 11-19 times; 20-30 times; 31-99 times; 100 or more times]

Participants who indicated ever using any type of e-cigarette were asked:

First use

When you used an electronic cigarette/e-cig/vape pen/vapor pens for the first time, which of the following did you use? [Response choices: Disposable/single use E-Cigarettes, Rechargeable cigarette-shaped, Larger than a cigarette, Large size "tank" device, Hookah pens, Vape pens, Mods, JUULs, Other/Don't know]

Did the first e-cigarette/vape you used contain NICOTINE? [Response choices: yes, no, unsure]

Was the FIRST [e-cigarette/vape, JUUL] you used flavored? [Response choices: yes, no]

Current use

During the LAST 30 DAYS, ON ABOUT HOW MANY DAYS did you smoke/use… [an e-cigarette, JUUL, a cigarette] (Put a 0 if you did not use the product in the last 30 days). [Response: number of days 0 to 30]

In the LAST 7 DAYS, ON ABOUT HOW MANY DAYS did you smoke/use... [an e-cigarette, JUUL, a cigarette] (Put a 0 if you did not use the product in the last 7 days) [Response: number of days 0 to 7].

© 2018 McKelvey K et al. *JAMA Network Open.*

Flavors

Participants who indicated ever-use of JUUL-style e-cigarette were asked: What flavor was the first JUUL you used? [Response choices for JUUL: Not sure/Don't remember, Mango, Cool Mint, Virginia Tobacco, Fruit Medley, Creme Brulee, Cool Cucumber, Classic Tobacco, Cool Menthol, Other (fill in)]

Participants who indicated ever-use of other e-cigarettes/vapes were asked: What flavor was the FIRST e-cigarette/vape you used? [Response choices for other e-cigarettes: Not sure/Don't remember, Tobacco flavored, Mint, Wintergreen, Menthol, Fruit (e.g., cherry, blueberry, strawberry, watermelon, coconut), Coffee (coffee or any related flavor – e.g., espresso, latte, cappuccino, etc.), Candy or dessert flavors (e.g., caramel, vanilla, chocolate, ice cream, mud pie), Spice (e.g., clove, cinnamon, nutmeg), Alcohol or cocktail (e.g., wine, bourbon, rum, brandy, tequila, whiskey, beer, mai-tai, daiquiri), Other (fill in)]

Perceptions

Social norms of JUUL and e-cigarettes:

Out of 5 of your CLOSEST FRIENDS, how many have EVER TRIED [an e-cigarette, JUUL]? [Response: between 0 and 5]

OUT OF 5 OF your CLOSEST FRIENDS, how many have USED [an e-cigarette, JUUL]? [Response: between 0 and 5]

IN THE PAST 30 DAYS? OUT OF 5 OF your CLOSEST FRIENDS, how many have USED [an e-cigarette, JUUL] REGULARLY? [Response: between 0 and 5]

Social acceptability of JUUL and e-cigarettes:

FRIENDS think it is ok (socially acceptable) for me to TRY just once [an e-cigarette, JUUL]? [Response choices: strongly disagree, disagree, agree, strongly agree]

FRIENDS think it is ok (socially acceptable) for me to USE ONCE IN A WHILE [an e-cigarette, JUUL]. [Response choices: strongly disagree, disagree, agree, strongly agree]

FRIENDS think it is ok (socially acceptable) for me to USE REGULARLY [an e-cigarette, JUUL]. [Response choices: strongly disagree, disagree, agree, strongly agree]

© 2018 McKelvey K et al. *JAMA Network Open.*

Perceived Prevalence

Out of 100 TEENS/YOUNG ADULTS YOUR AGE, how many do you think HAVE TRIED… [an e-cigarette, JUUL] [Response: number out of 100]

Out of 100 TEENS/YOUNG ADULTS YOUR AGE, how many do you think HAVE USED IN THE PAST 30 DAYS… [an e-cigarette, JUUL] [Response: number out of 100]

Out of 100 TEENS/YOUNG ADULTS YOUR AGE, how many do you think USE [an e-cigarette, JUUL] REGULARLY? [Response: number out of 100]

Short term risks or benefits

Participants first read the following scenario: Whether or not you have used any of these products, imagine that you JUST BEGAN USING [an e-cigarette, JUUL]. You use it about 2 to 3 times a day, every day. Sometimes you use it alone and sometimes you use it with friends. If you use the product 2 to 3 times each day, what is the chance that ...[Response: 0-100%]:

- You'll get a bad cough,
- You'll have trouble catching your breath,
- You'll have better concentration,
- Your friends will be upset with you,
- You'll feel less stressed,
- You'll get into trouble,
- You'll look cool,
- You'll become addicted to the product,
- You'll feel high or buzzed,
- You'll get lung disease (COPD),
- You'll feel less anxious,
- You'll feel less depressed.

Perceived long term risks

© 2018 McKelvey K et al. *JAMA Network Open.*

Participants first read the following scenario: Imagine now that you CONTINUE TO USE [an e-cigarette, JUUL] 2 to 3 times a day, every day FOR THE REST OF YOUR LIFE. What is the chance that… [Response: 0 to 100%]

- You'll get oral (mouth) cancer,
- You'll have a heart attack,
- You'll get lung cancer,
- You'll get another tobacco-related disease,
- You'll get wrinkles on your face,
- You'll die from a tobacco-related disease,
- You'll feel less anxious,
- You'll feel less depressed.

Addiction or Nicotine Dependence

Participants were asked the following 10 questions, which constitute the Hooked On Nicotine Checklist. [Response choices for all: yes, no]

Have you ever TRIED TO QUIT E-cigarettes/vapes (not including JUULs) or JUULs below but couldn't?

Do you use E-cigarettes/vapes (not including JUULs) or JUULs now because it is really hard to quit?

Have you ever FELT LIKE YOU WERE ADDICTED to using E-cigarettes/vapes (not including JUULs) or JUULs?

Have you ever felt like you REALLY NEEDED E-cigarettes/vapes (not including JUULs) or JUULs?

Do you ever have STRONG CRAVINGS for E-cigarettes/vapes (not including JUULs) or JUULs?

© 2018 McKelvey K et al. *JAMA Network Open.*

Is it hard to keep from using E-cigarettes/vapes (not including JUULs) or JUULs where you are not supposed to (like in school)?

If you have tried to stop using e-cigarettes/vapes (not including JUULs) or JUULs or if you have not used these product(s) for a while:

Did you find it hard to concentrate because you could not use E-cigarettes/vapes (not including JUULs) or JUULs?

Did you feel more irritable because you could not use E-cigarettes/vapes (not including JUULs) or JUULs?

Did you feel a strong need or urge to use any of E-cigarettes/vapes (not including JUULs) or JUULs?

Did you feel nervous, restless, or anxious because you couldn’t use E-cigarettes/vapes (not including JUULs) or JUULs?

© 2018 McKelvey K et al. *JAMA Network Open.*
